# QuantiFERON-TB Gold Plus Conversion Among Healthcare Workers: A Retrospective Cohort Study on Occupational and Metabolic Factors

**DOI:** 10.3390/healthcare14142221

**Published:** 2026-07-22

**Authors:** Stefano Barnabei, Giuseppina Somma, Stella Andreadi, Andrea Magrini, Emiliano Santacroce, Cristiana Ferrari, Lorenzo Ippoliti, Luca Coppeta

**Affiliations:** 1 Department of Biomedicine and Prevention, University of Rome Tor Vergata, 00133 Rome, Italy; stefano.barnabei@ptvonline.it (S.B.); giuseppina.somma@ptvonline.it (G.S.); stella.p.and@gmail.com (S.A.); andrea.magrini@uniroma2.it (A.M.); luca.coppeta@uniroma2.it (L.C.); 2 Workplace Prevention and Safety Service, Local Sanitary Unit Roma 5, 00012 Guidonia Montecelio, Italy; emiliano.santacroce@aslroma5.it; 3 PhD Program in Social, Occupational and Medico-Legal Sciences, Department of Biomedicine and Prevention, University of Rome Tor Vergata, 00133 Rome, Italy; 4 Faculty of Medicine, Saint Camillus International University of Health Sciences, 00131 Rome, Italy

**Keywords:** tuberculosis infection, QuantiFERON-TB Gold Plus, healthcare workers, occupational exposure, QuantiFERON conversion, lipid metabolism, hypercholesterolemia

## Abstract

**Background:** Healthcare workers (HCWs) remain at risk of occupational exposure to *Mycobacterium tuberculosis*. Although workplace-related risk factors have been extensively investigated, the contribution of host metabolic factors to tuberculosis infection (TBI) susceptibility remains poorly understood. This study evaluated QuantiFERON-TB Gold Plus (QFT-Plus) conversion and its association with demographic, occupational, and metabolic risk factors. **Methods:** A retrospective cohort study was conducted among 3309 HCWs undergoing annual routine occupational health surveillance at Policlinico Tor Vergata in Rome (Italy) from 2017 to 2025. Data included age, sex, job category, night-shift work, departmental risk level, and biochemical parameters (lipid profile, blood glucose, and vitamin D levels). Associations with QFT-Plus conversion were assessed using univariate analyses, binary logistic regression, and multivariate Cox regression models. **Results:** During follow-up, 87 participants (2.6%) experienced QFT-Plus conversion. In univariate analysis, age ≥ 40 years (3.98% vs. 1.50%; *p* < 0.001) and hypercholesterolemia (3.8% vs. 2.1%; *p* = 0.004) were significantly associated with conversion. However, neither variable remained statistically significant in multivariate analyses (age: *p* = 0.734; hypercholesterolemia: *p* = 0.834). Occupational factors, including job category, night-shift work, and assignment to high-risk departments, were not significantly associated with QFT-Plus conversion. Likewise, hyperglycemia and vitamin D deficiency showed no significant associations. **Conclusions:** QFT-Plus conversion incidence was low, supporting the effectiveness of current hospital infection control measures in this low-incidence setting. No demographic, occupational, or metabolic factor emerged as an independent predictor of conversion. These findings should be interpreted considering the limited number of conversion events and the multifactorial nature of TBI susceptibility.

## 1. Introduction

According to the latest World Health Organization (WHO) Global Report, tuberculosis (TB) is one of the leading causes of death worldwide. In 2024, approximately 10.7 million people fell ill with TB, and 1.23 million people died from this infectious disease. Additionally, nearly a quarter of the global population has tuberculosis infection (TBI), which is the main source of new cases of active disease [1]. TBI, previously referred to as latent tuberculosis infection (LTBI), is characterized by a persistent immune response to *Mycobacterium tuberculosis* antigens in the absence of clinical signs or symptoms of the disease. Thus, the early identification and prophylactic treatment of individuals with TBI are key to TB control and elimination efforts [2,3].

The WHO classifies Italy as a low-incidence country. According to the Italian National Institute of Health, the TB incidence rate in 2024 was approximately 5.3 cases per 100,000 people, with an uneven distribution and a higher concentration among specific high-risk groups [4]. Healthcare workers (HCWs) are particularly at risk due to frequent contact with potentially infected patients in hospital settings. Additionally, HCWs may acquire TB infection outside the workplace, potentially transmitting it to colleagues and patients [2].

Despite the adoption of preventive measures, such as the use of personal protective equipment (PPE), the implementation of isolation protocols, and occupational health surveillance programs, the risk of occupational exposure to *Mycobacterium tuberculosis* remains significant, especially in high-burden healthcare settings [5].

In recent years, research has increasingly focused on factors beyond traditional occupational exposure, highlighting the potential role of individual characteristics, particularly metabolic and inflammatory conditions, in influencing susceptibility to TB infection. Several studies suggest that hypercholesterolemia may promote the persistence of dormant mycobacteria [6,7]; hyperglycemia and diabetes mellitus have been associated with an increased risk of developing active TB, poorer clinical outcomes, and higher bacterial burden [8]; and vitamin D deficiency appears to negatively affect the host immune response, being associated with both an increased risk of active disease and tuberculin skin test or QuantiFERON conversion [9,10,11].

However, data regarding the potential role of metabolic factors in QuantiFERON-TB Gold-Plus (QFT-Plus) conversion among apparently healthy healthcare workers undergoing occupational surveillance are limited. In particular, evidence on the association between metabolic parameters and QFT-Plus conversion in longitudinal occupational health settings remains scarce, and available studies have mainly focused on active tuberculosis or cross-sectional IGRA outcomes rather than incident immunological conversion. This leaves an important gap in understanding the early dynamics of tuberculosis infection in healthcare worker populations.

In this context, conversion of immunological tests for TBI, such as the QuantiFERON test (interferon-gamma release assay, IGRA), is a relevant indicator of new TBI and a useful tool in occupational health surveillance programs for healthcare workers.

This study aimed to evaluate the use of QFT-Plus conversion as a marker of new tuberculosis infection among healthcare workers at Policlinico Tor Vergata (PTV) in Rome. The study examined the relationship between this marker and occupational, metabolic, and demographic risk factors.

## 2. Materials and Methods

A retrospective cohort study was conducted on 3309 healthcare workers and employees of the Policlinico Tor Vergata (PTV) in Rome, who underwent periodic occupational health surveillance and screening for *Mycobacterium tuberculosis* infection using the LIAISON QuantiFERON-TB Gold Plus (QFT-Plus) interferon-gamma (IFN-γ) release assay (IGRA), manufactured by DiaSorin S.p.A., Saluggia, Italy.

The study was based on clinical and laboratory data collected by the PTV Occupational Medicine Unit. The analyzed sample included medical doctors, nurses, laboratory technicians, radiology technicians, and administrative staff.

The following variables were collected for each participant: age, sex, occupational role, night shift work, hospital department TB risk classification, serum lipid profile (total cholesterol, HDL, LDL, and triglycerides), blood glucose levels, serum vitamin D levels, and QFT-Plus results.

The age cut-off of 40 years was used to distinguish between younger and older healthcare workers, reflecting potential differences in cumulative occupational exposure.

Hospital departments were classified according to TB exposure risk, as defined by Italian Ministry of Health guidelines, which categorize departments into one of five increasing-risk groups (A-E). In the present study, no departments were classified as category E; departments classified as C and D were considered high-risk settings for TB transmission [12]. Classification was applied by the PTV Occupational Medicine Unit based on the type of clinical activity and the estimated frequency of exposure to patients with suspected or confirmed tuberculosis. Specifically, departments with higher patient turnover and a greater likelihood of exposure to airborne infectious diseases (e.g., internal medicine, pulmonology, infectious diseases, and emergency departments) were classified as high-risk settings.

QFT-Plus conversions were assessed retrospectively over the surveillance period from 2017 to 2025. QFT-Plus results were interpreted according to the manufacturer’s standard cut-off, with interferon-gamma (IFN-γ) values ≥ 0.35 IU/mL considered positive. For the present analysis, only the qualitative laboratory interpretation (positive/negative) reported in the occupational health records was used. Quantitative IFN-γ values were not extracted from the laboratory reports, and borderline results or reversions were not specifically evaluated. QFT-Plus conversion was defined as transitioning from a negative to a positive QFT-Plus result during follow-up. Previous records were reviewed for subjects with a positive QFT-Plus result in 2025 to identify conversions that occurred during the surveillance period.

All healthcare workers underwent annual QFT-Plus testing as part of the routine occupational health surveillance program. Follow-up time was calculated on an annual basis up to 2025. For subjects with documented QFT-Plus conversion, the last negative result and the first positive result were identified retrospectively, and the conversion event was assigned to the year of the first positive QFT-Plus result. Participants with negative QFT-Plus results in 2025 were classified as non-converters during the study period.

The biochemical variables were categorized according to the PTV Chemical Analytical Laboratory’s reference cut-off values as follows: hypercholesterolemia (total cholesterol ≥ 200 mg/dL), hyperglycemia (blood glucose ≥ 100 mg/dL), and vitamin D deficiency (vitamin D level < 30 ng/mL). For hyperglycemia, the threshold of 100 mg/dL was chosen to capture early alterations in glucose metabolism, including both impaired fasting glucose and diabetes, in order to better reflect the full spectrum of metabolic risk in the study population. Biochemical measurements were obtained from the most recent occupational health surveillance assessment available for each participant in 2025. No missing data were present for the biochemical variables included in the analysis.

Statistical analyses were performed using IBM SPSS Statistics version 28.0. Continuous variables were compared using a Student’s *t*-test, and categorical variables were analyzed using a chi-square test.

We evaluated the association between QFT-Plus conversion and occupational, metabolic, and demographic risk factors using binary logistic regression. The independent variables included age, sex, professional role, night shift work, hospital department TB risk classification, hyperglycemia, hypercholesterolemia, and vitamin D deficiency.

Additionally, a multivariate Cox regression analysis was performed to evaluate the risk of QFT-Plus conversion among population subgroups according to these characteristics. Follow-up time was reconstructed from annual occupational health surveillance data. Participants contributed person-time from their first available negative QFT-Plus result recorded during occupational surveillance until the first positive QFT-Plus result or the end of the surveillance period in 2025. Because QFT-Plus testing was conducted annually, the exact date of conversion could not be determined; therefore, the event was assigned to the year of the first positive result. The Cox regression model estimated adjusted hazard ratios (HRs) with corresponding 95% confidence intervals (95% CI) to assess the independent association between the exposure variables and conversion. The multivariate model included all variables evaluated in the logistic regression analysis.

A *p*-value of less than 0.05 was considered statistically significant.

## 3. Results

The study population consisted of 3309 healthcare workers: of those, 1167 were males (35.3%) and 2142 were females (64.7%). The mean age of the study population was 40.7 ± 11.7 years. The mean serum total cholesterol and blood glucose levels were 185.1 ± 34.8 mg/dL and 89.7 ± 12.0 mg/dL, respectively. Among the study cohort, 91 HCWs had a positive QFT-Plus result in 2025. Retrospective review of occupational health records identified 87 QFT-Plus conversion events occurring during the 2017–2025 study period, accounting for 2.6% of the cohort. The remaining four subjects had documented positive QFT-Plus results before 2017 and were therefore not classified as incident QFT-Plus conversion cases. The conversion incidence rate was 3.96 new cases per 1000 person-years. The median follow-up duration was 8 years, ranging from 1 to 8 years. The mean follow-up was 6.64 person-years. The main characteristics of the study population are reported in Table 1.

In terms of age distribution, 1506 participants (45.5%) were 40 years of age or older, while 1803 (54.5%) participants were younger than 40 years of age. The QFT-Plus conversion rate was higher among subjects aged 40 years and older compared with younger workers (3.98% vs. 1.50%, respectively). Univariate analysis revealed a significant association between age and conversion (*p* < 0.001). However, this association was not significant in the multivariate Cox regression model (*p* = 0.734).

There was no statistically significant association observed between sex and QFT-Plus conversion (*p* = 0.598). The conversion rate was 2.5% among female healthcare workers and 2.8% among males.

Additionally, work-related variables, such as occupational role (nurses vs. other HCWs, *p* = 0.659), night shift work (*p* = 0.235), and assignment to high-risk hospital departments (*p* = 0.614), were not significantly associated with conversion.

Among the metabolic variables examined, hypercholesterolemia was significantly associated with QFT-Plus conversion in the univariate analysis (*p* = 0.004). A higher proportion of conversions was observed among subjects with elevated cholesterol levels compared to normocholesterolemic individuals (3.8% vs. 2.1%). However, this association was not confirmed in the multivariate analysis (*p* = 0.834), which suggests an absence of an independent effect.

Univariate analysis revealed no statistically significant associations for hyperglycemia (*p* = 0.17) or vitamin D deficiency (*p* = 0.675). The main results of the univariate logistic regression and multivariate Cox regression analyses, including odds ratios (ORs), hazard ratios (HRs), 95% confidence intervals (95% CIs), and corresponding *p*-values, are presented in Table 2.

**Figure 1:**
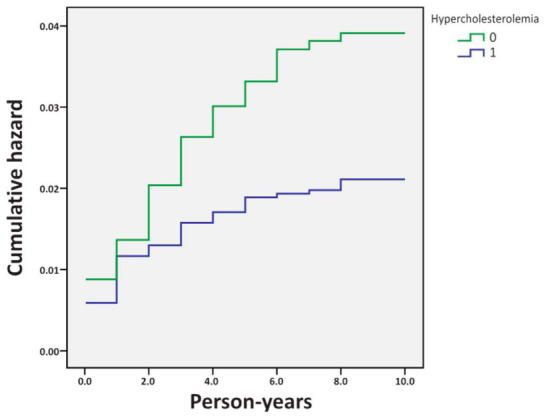
Cox model-based cumulative hazard function according to hypercholesterolemia status.

We used Cox model-based cumulative hazard functions to compare subjects with and without hypercholesterolemia. As shown in Figure 1, hypercholesterolemic individuals exhibited a higher cumulative hazard of QFT-Plus conversion over time compared with normocholesterolemic subjects.

## 4. Discussion

The present study aimed to evaluate the association between occupational, metabolic, and demographic factors and QFT-Plus conversion among healthcare workers undergoing occupational surveillance. Overall, no variable emerged as an independent predictor of conversion in the multivariate analysis. However, this finding should be interpreted cautiously given the limited number of conversion events.

The study revealed a low QFT-Plus conversion rate of 2.6% of the study population, corresponding to an incidence rate of 3.96 new cases per 1000 person-years. These results are consistent with previous reports from countries with low rates of tuberculosis, such as Italy, where HCWs show similarly low conversion rates despite persistent occupational exposure [13]. With only 87 conversions across multiple covariates, the regression models may have lacked the power to detect weak but genuine associations. Accordingly, given the limited number of events, modest associations may have remained undetected in the multivariate analysis. The low incidence observed likely reflects the effectiveness of current infection control and occupational surveillance strategies. Nevertheless, the occurrence of conversion confirms that occupational exposure to *Mycobacterium tuberculosis* persists, although at low levels.

Univariate analysis identified significant associations with age and hypercholesterolemia, suggesting that both cumulative exposure and host metabolic factors may contribute to susceptibility to tuberculosis infection.

Among the metabolic factors examined, hypercholesterolemia was significantly associated with conversion in the univariate analysis, with affected individuals showing higher conversion rates than those without hypercholesterolemia. However, this association was not confirmed after adjustment, which suggests that confounding factors may have influenced the results rather than hypercholesterolemia having an independent effect on QFT-Plus conversion.

Nevertheless, previous experimental studies suggest biologically plausible mechanisms that may underlie the observed univariate association. There is increasing evidence supporting a central role of lipid metabolism in the host–pathogen interactions in tuberculosis. *Mycobacterium tuberculosis* can exploit host lipids as an energy source to facilitate intracellular survival and long-term persistence during latent infection [7]. Experimental studies in hypercholesterolemic animal models, such as ApoE-deficient mice, have demonstrated increased susceptibility to mycobacterial infection and enhanced inflammatory responses compared with controls [14].

Furthermore, lipid accumulation within macrophages contributes to the formation of foam cells, which creates a microenvironment that permits mycobacterial persistence within granulomatous lesions. While these mechanisms may partially explain the observed association, causality cannot be established. The lack of significance in the multivariate analysis likely reflects the multifactorial nature of QFT-Plus conversion, in which metabolic alterations interact with age, the duration of occupational exposure, immune status, and other unmeasured factors. Additionally, total cholesterol alone may not adequately capture the complexity of lipid-related immune dysregulation. In this context, active tuberculosis has been increasingly recognized as a systemic disorder where metabolic alterations are tightly linked with complex immune and inflammatory-driven cascades, including hypercoagulability states that alter the vascular microenvironment [15].

Accordingly, these findings do not support an independent association between hypercholesterolemia and QFT-Plus conversion.

Hyperglycemia did not show a statistically significant association with QFT-Plus conversion in either univariate or multivariate analysis. This finding contrasts with previous evidence reporting an increased susceptibility to tuberculosis infection among patients with diabetes mellitus [8,16].

Several mechanisms have been proposed to explain the relationship between diabetes and tuberculosis, including impaired macrophage activation, reduced T-cell-mediated immunity, chronic low-grade inflammation, and altered cytokine responses. Diabetic patients have also been shown to have a higher mycobacterial burden, delayed sputum conversion, and poorer clinical outcomes [8].

In the present study, hyperglycemia was evaluated using a blood glucose threshold of ≥100 mg/dL. This threshold does not necessarily identify subjects with overt diabetes mellitus or with the metabolic consequences typically associated with the disease. Since diabetes mellitus is usually diagnosed at blood glucose levels of at least 126 mg/dL, the study population likely included individuals with mild glycemic alterations or prediabetes rather than subjects with clinically significant metabolic disease. This could reduce the ability to detect significant associations.

Vitamin D deficiency showed a borderline association in the multivariable Cox model, but this did not reach statistical significance and should be interpreted cautiously.

In univariate analysis, age was significantly associated with QFT-Plus conversion, with higher conversion rates observed in older healthcare workers. This result may reflect cumulative occupational exposure over time, as well as age-related changes in immune function. In particular, immunosenescence could contribute to a less efficient host immune response against intracellular pathogens, including *Mycobacterium tuberculosis*, thereby increasing susceptibility to infection [17].

Interestingly, occupational variables that are traditionally considered relevant to tuberculosis exposure, such as assignment to high-risk departments and working the night shift, were not significantly associated with QFT-Plus conversion. This finding should be interpreted with caution in light of the exposure assessment used in the present study. Occupational exposure was assessed using relatively broad indicators, including job category, night-shift work, and departmental risk classification, which may not fully capture the variability in tuberculosis exposure experienced by individual healthcare workers, potentially leading to exposure misclassification. In low-incidence settings such as Italy, the absence of significant associations may also reflect the effectiveness of preventive measures or the study’s limited statistical power, as previously discussed. Current tuberculosis prevention strategies implemented within the hospital setting, including respiratory isolation protocols, the use of personal protective equipment, and regular occupational health surveillance programs, may have reduced occupational transmission [2,3].

Additionally, occupational exposure to *Mycobacterium tuberculosis* may be less intense and more varied in low-incidence countries than in high-burden settings, which reduces the measurable impact of workplace-related risk factors on QFT-Plus conversion [5,13]. These findings further support the importance of maintaining strict infection control measures even in low-incidence healthcare settings.

This study has several limitations that should be considered when interpreting the findings. First, its retrospective design relied on routinely collected occupational health surveillance data, limiting the availability of detailed information on potential confounding factors including lifestyle habits such as smoking, body mass index, comorbidities, systemic inflammatory status, immunosuppressive conditions, and pharmacological treatments. Consequently, the ability to fully adjust for all variables potentially associated with QFT-Plus conversion may have been reduced.

Second, the heterogeneous age distribution of the study population reflects the real-world composition of the healthcare workforce and represents an inherent characteristic of occupational surveillance datasets. To mitigate its potential confounding effect, age was included as a covariate in the multivariate regression models. Nevertheless, given the observational and retrospective nature of the study, residual confounding related to age cannot be completely excluded.

Third, QFT-Plus conversion was used as a surrogate marker of *Mycobacterium tuberculosis* infection. Although widely adopted in occupational health surveillance and epidemiological studies of TBI, QFT-Plus conversion does not perfectly reflect true infection events. The assay is subject to intrinsic limitations in sensitivity and may be affected by the timing of exposure and testing. In particular, recent infections may not yet be detectable at the time of follow-up testing, while exposures occurring after the last negative test may fall outside the observational period. Furthermore, longitudinal variability in QFT-Plus results, including possible spontaneous reversion, together with the lack of extracted quantitative IFN-γ data and the absence of evaluation of borderline results, may contribute to outcome misclassification.

Despite these limitations, QFT-Plus conversion remains one of the most widely accepted proxy measures for recent tuberculosis infection in healthcare settings and has been extensively used in studies assessing TBI incidence among healthcare workers [18]. Moreover, available evidence suggests that spontaneous reversion rates are generally low in longitudinal occupational cohorts, supporting the utility of this marker for epidemiological surveillance [19].

Regarding the timing of exposure and potential delayed test conversion, it is theoretically possible that a small number of individuals who were recently infected may not have been identified during the observation period, as immunological conversion may occur several weeks after exposure [20]. However, given the low cumulative incidence observed in the study population, the number of potentially missed conversions is expected to be minimal and unlikely to have materially influenced the overall findings.

Fourth, the study dataset did not include information on prior occupational or community exposure to *Mycobacterium tuberculosis*, limiting the ability to fully reconstruct individual exposure histories. However, this limitation is partially mitigated by the presence of a structured occupational health surveillance system that includes post-exposure monitoring procedures. During the study period, no QFT-Plus conversions were documented within post-exposure occupational follow-up pathways, suggesting that the impact of unavailable exposure information on the study outcomes was likely limited.

Nevertheless, the large study population and the use of a standardized occupational surveillance protocol strengthen the consistency and reliability of the overall findings.

Despite its limitations, this study provides additional data on the potential relationship between host metabolic status and QFT-Plus conversion among healthcare workers. Although no independent associations were identified after adjustment for potential confounders, the observed univariate findings, together with the biological plausibility of the underlying mechanisms, support the need for further prospective investigations incorporating comprehensive metabolic, immunological, and lifestyle assessments. Such studies are necessary to better elucidate the contribution of host metabolic factors to susceptibility to tuberculosis infection and their interaction with occupational exposure in low-incidence healthcare settings.

## 5. Conclusions

In the present study, we found a low incidence of QFT-Plus conversion (3.96 cases per 1000 person-years) among healthcare workers under routine occupational surveillance, regardless of occupational TB risk level, supporting the effectiveness of current infection control measures in preventing nosocomial transmission of *Mycobacterium tuberculosis*. Although no variable emerged as an independent predictor of conversion at multivariate analysis, univariate findings for older age and hypercholesterolemia suggest a possible role of cumulative exposure and host lipid metabolism in TBI susceptibility, warranting further investigation through larger prospective studies with comprehensive metabolic and immunological assessments.

In conclusion, our findings support the need for continued surveillance, emphasizing the central role of occupational medicine in hospital-based TB monitoring programs.

Further prospective studies are needed to determine whether specific risk profiles could inform future screening approaches.

## Figures and Tables

**Table 1:** Main characteristics of the study population.

Variable	Study Population (N = 3309)
**Age, years**	40.7 ± 11.7
Age ≥ 40 years old	1506 (45.5%)
Age < 40 years old	1803 (54.5%)
**Sex**	
Female	2142 (64.7%)
Male	1167 (35.3%)
**Biochemical variables**	
Total cholesterol, mg/dL	185.1 ± 34.8
Blood glucose, mg/dL	89.7 ± 12.0
Hypercholesterolemia	1046 (31.6%)
Hyperglycemia	411 (12.4%)
Vitamin D deficiency	2368 (71.6%)
**QFT-Plus conversion**	87 (2.6%)
Incidence rate	3.96 per 1000 person-years

**Table 2:** Factors associated with QFT-Plus conversion: univariate logistic regression and multivariable Cox regression analyses.

Variables	QFT Conversion %	QFT Non-Conversion %	Univariate Logistic Regression OR (95% CI)	*p* Value Univariate	Multivariate Cox Regression HR (95% CI)	*p* Value Multivariate
**Male Sex**	1.00%	34.27%	1.125 (0.725−1.745)	0.598	1.596 (0.447−5.697)	0.472
**Age ≥ 40 years**	1.81%	43.67%	2.733 (1.726−4.327)	<0.001	1.284 (0.305−5.410)	0.734
**Hyperglycemia**	0.45%	11.97%	1.487 (0.844−2.619)	0.17	0.979 (0.198−4.837)	0.979
**Hypercholesterolemia**	1.21%	30.40%	1.875 (1.222−2.877)	0.004	1.143 (0.329−3.970)	0.834
**Vitamin D deficiency**	1.93%	69.63%	1.109 (0.684−1.796)	0.675	0.294 (0.085−1.013)	0.052
**Night Shift Work**	1.47%	69.44%	0.482 (0.144−1.610)	0.235	0.401 (0.113−1.427)	0.158
**Job (Nurse)**	0.98%	29.34%	1.324 (0.380−4.607)	0.659	1.264 (0.332−4.811)	0.732
**High-risk Department**	2.20%	73.35%	1.490 (0.317−7.012)	0.614	2.144 (0.414−11.094)	0.363

Percentages refer to the total study population (*n* = 3309).

## Data Availability

The data presented in this study are available on request from the corresponding author due to privacy and ethical considerations, as the dataset includes sensitive occupational and health-related information of healthcare workers.

## Abbreviations

The following abbreviations are used in this manuscript:

HCWs	Healthcare workers
TBI	Tuberculosis infection
QFT-Plus	QuantiFERON-TB Gold Plus
TB	Tuberculosis
IGRA	Interferon-gamma release assay
PPE	Personal protective equipment
